# Dressed Photons Induced Resistance Oscillation and Zero Resistance in Arrayed Simple Harmonic Oscillators with No Impurity

**DOI:** 10.1038/srep37763

**Published:** 2016-11-25

**Authors:** Chih-Chun Chang, Guang-Yin Chen, Lee Lin

**Affiliations:** 1 Department of Physics, National Chung-Hsing University, Taichung 402, Taiwan

## Abstract

We investigate a system of an array of *N* simple harmonic oscillators (SHO) interacting with photons through QED interaction. As the energy of photon is around the spacing between SHO energy levels, energy gaps appear in the dispersion relation of the interacted (dressed) photons. This is quite different from the dispersion relation of free photons. Due to interactions between dressed photonic field and arrayed SHO, the photoresistance of this system shows oscillations and also drops to zero as irradiated by EM field of varying frequencies.

Within the last 20 years, a growing number of researches have been conducted to explore interactions between atoms and photonic field^1^,^2^,^3^,^4^,^5^ for the purpose of investigating fundamental physics, and practical applications. Since their discoveries around 2002^1^,^2^, the phenomena of microwave-induced zero resistance (MIZR), and microwave-induced resistance oscillation (MIRO) in two-dimensional electron gas (2DEG) have attracted the interests of many scientists. According to experimental data^1^,^2^,^6^,^7^,^8^,^9^,^10^,^11^,^12^,^13^,^14^,^15^,^16^,^17^,^18^,^19^, the magneto-resistance of the two-dimensional semiconductor shows peculiar oscillation with the irradiation of microwave on the sample. Many theories have been proposed for the MIZR and MIRO in 2DEG^6^,^20^,^21^,^22^,^23^,^24^,^25^,^26^,^27^,^28^,^29^,^30^,^31^,^32^,^33^,^34^,^35^,^36^,^37^,^38^,^39^,^40^,^41^,^42^,^43^,^44^,^45^,^46^,^47^,^48^,^49^,^50^,^51^,^52^,^53^,^54^,^55^,^56^. Among these researches, the displacement model of the photon assisted impurity and phonon scatterings has been studied in many works, and several accomplishments have been achieved in these theories. In these works, impurities in the samples seem to play important roles for the transportation of electrons.

On the other hand, as samples become purer, some experiments^1^,^2^ show that MIZR & MIRO still exist and are not less apparent. And people may wonder if MIZR & MIRO can occur in *pure* systems due to some other mechanisms. We would propose in this paper a one-dimensional system of pure arrayed simple harmonic oscillator (SHO) with no phonons at zero temperature. It can be exhibited that MIZR & MIRO can occur due to quantum electrodynamical (QED) interaction between photonic field and arrayed SHO.

The microwave irradiated 2DEG in a magnetic fied is a system with energy levels of equal energy spacing as Landau levels. Considering such a significant feature of the irradiated 2DEG system in a magnetic field, for simplicity of analytical calculations, we consider an array of *N* SHO interacting with a photonic field generated from an external source. The SHO states can be changed by emission and absorption of photons. When the photons are absorbed by the arrayed SHO, they are annihilated and this would have great impact on the quantum states of photonic field such that its dispersion relation would be different from that of free photons. As the energy of photon is around the spacing between SHO energy levels, the photon will be absorbed and is not in the propagating mode but the attenuated mode. Therefore, energy gaps appear in the dispersion relation of the interacted (dressed) photon. Due to close interactions between photonic field and arrayed SHO, the influences of the dressed photonic fields to atomic states and associated physical behaviors of the arrayed SHO will be quite different from those coming from free photonic fields. And we would report in this paper that the photoresistance of this system shows oscillations and also drops to zero as irradiated by EM field of varying frequencies.

The structure of this paper is as follows. In the Results section, we started with the Hamiltonians of photonic field in one dimension, an array of *N* SHO with hoppings, and the interaction between them through QED coupling. In the subsections of Electron propagator & Photon propagator, we calculate the dressed propagators of electrons and photons, respectively. Direct current (DC) conductivity of the irradiated SHO is obtained through these (dressed) propagators, and is presented in the subsection of DC conductivity. Comparisons with experimental works are mentioned. Summaries and discussions will follow in the Discussion section. Photonic dispersion relation and Bloch’s wave function for photons are shown in the Methods section.

## Results

We consider a model of a pure system of *N* SHO on a linear lattice (*x*-direction) interacting with a quantized EM field 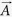 through the QED coupling. To avoid unnecessary complications, we restrict our studies in zero temperature throughout this paper. Assuming that the EM wave is moving in the *x*-direction and uniform along 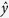, 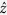, we can then write the vector potential 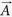 as 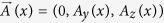, by adopting radiation gauge (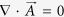). The Hamiltonian *H*_*em*_ for the EM field is


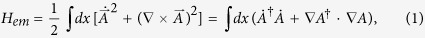


where *field operators A(x*) & *A*^†^(*x*) are so defined 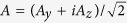, 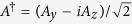 which describe annihilation and creation of one photon, respectively. The Hamiltonian of an array of *N* SHO with hoppings to neighboring sites is


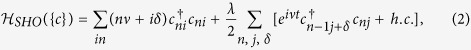


and the interaction between the photonic field and the SHO array is


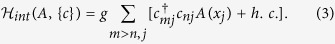


Here the field operator (and its Hermitian conjugate (*h*.*c*.)) of electron associated with the SHO at site *i* on the *n*-th energy level is denoted as *c*_*ni*_ (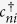) with *ν* the energy spacing between two adjacent levels (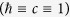. And we follow the selection rule in the original irradiated 2DEG system in a magnetic field^20^,^54^ by taking the inter-SHO-level transitions to be *n* ↔ *n* ± 1 for hopping to its nearest-neighbor sites with 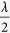 as hopping coefficient. And 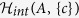 is the QED type Hamiltonian for the atom-photon interaction with the coupling constant 
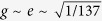
. In Eq. (2), *δ* is a positive (small) finite number and 1/*δ* is proportional to the relaxation time of the excited state. For simplicity, we assume *δ* a constant, and thus a uniform relaxation time for the system.

### Electron propagator

**Figure 1:**
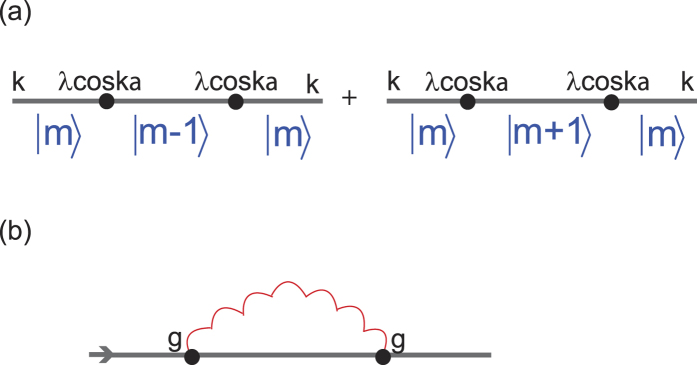
Self-mass. Effective masses of the propagator of electron due to hopping and atom-photon interaction are shown in (**a**) & (**b**), respectively.

In Eq. (2), written in the momentum space, the hopping term of the SHO (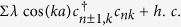) gives rise to an additional effective mass to the propagator of the electron on a typical energy level *m*, as is shown in Fig. 1(a),


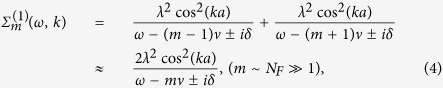


where *a* is the lattice spacing, the + (−) sign is for particle propagating forward (backward) in time; and the − sign propagator can be interpreted as the hole propagator. In obtaining the above equation, we have assumed that the electrons involving in the optical-electronic interactions are around the Fermi level *N*_*F*_.

Before interacting with photon, the propagator of the electron (with hopping taken into account) on a typical energy level *m*,


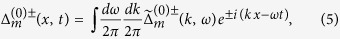


satisfies the following Dyson’s equation (by Eqs (2) and (4)),


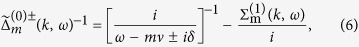


and can be solved as


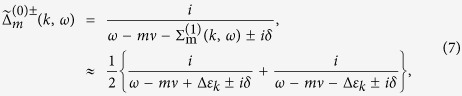


where


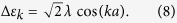


That is, the energy levels of the atoms are modified by 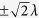 due to hopping. The additional effective mass of the propagator of the electron due to atom-photon interaction (Fig. 1(b)) is


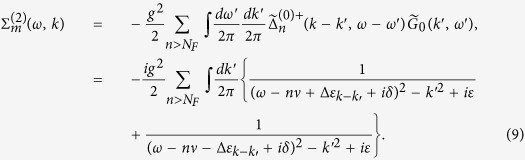


One thing needs to be noted is that because of hoppings and photon-electron interactions, the energy levels of the SHO would be modified (renormalized). The pole of the propagator of electron in the *n*-th level determines its dispersion relation *E*_*n*_(*k*). By Eqs (7)–(9), 22, 24, *E*_*n*_(*k*) ranges from 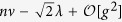 to 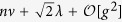. We can write 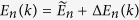, with 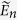 the minimum of *E*_*n*_(*k*) (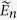 = *nν* − Δ*ν*, 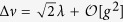) which is defined as the self-mass of electron in the *n*-th level, and the rest (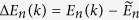) is the kinetic energy of it. Moreover, the self-masses of electrons are identified as the renormalized energy levels (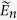
’s) of the arrayed SHO. In *d*-dimensions, Δ*ν* is expected to be 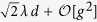.

### Photon propagator

**Figure 2:**
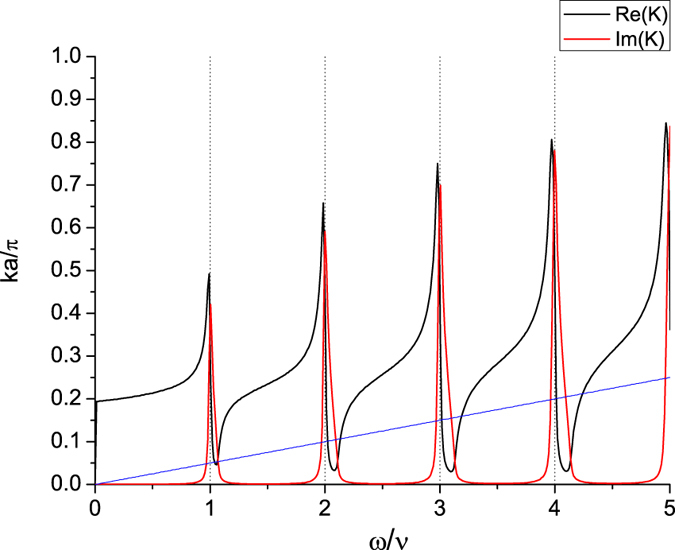
Dispersion. Dispersion relations of *k*_*ω*_*a*/*π*(≡*Re K*_*ω*_*a*/*π*) versus *ω*/*ν* [solid line], and *κ*_*ω*_*a*/*π*(≡*Im K*_*ω*_*a*/*π*) versus *ω*/*ν* [dashed line] of photonic field propagating in an array of SHO. The blue line represents the dispersion relation for free photon. Here we choose *g*^2^/*a* = 1/2, *ν* = *π*/20, *λ* = *ν*/10, *δ* = *ν*/200.

The Green’s function of the EM field *G(x*, *t*; *x*′, *t*′) satisfies the Dyson’s equation as,


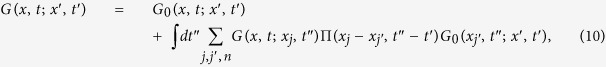


or can be expressed in the following way in the momentum space,


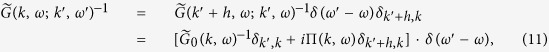


where 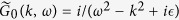 is the free propagator of the EM field (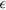 → 0^+^), *h*’s the reciprocal lattice vectors (*h* = 2*nπ*/*a*, *a* the lattice constant), and Π(*k*, *ω*) is


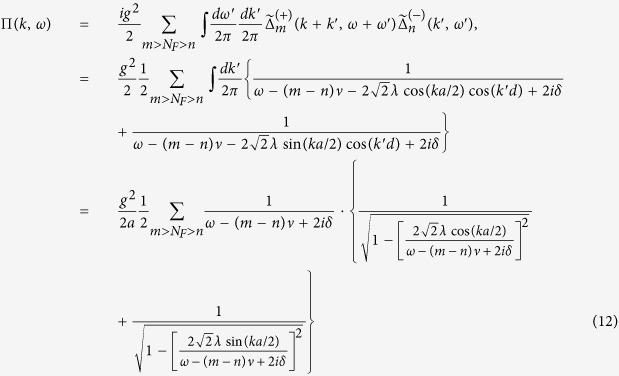


which represents the modification to the propagator (*self-energy*) of the EM wave due to atom-photon interaction, and it contains both real part and imaginary part originated from *δ*. The above running index *m (n*) for the electron (hole) propagator should be restricted by the condition *mν* − *ε*_*F*_ < *ω*_*s*_ (*ε*_*F*_ − *nν* < *ω*_*s*_), with *ω*_*s*_ the angular frequency of the applied EM field. From the Green’s function in Eq. (11), as is shown in the Methods section, the dispersion relation of the photon can be obtained (*c* ≡ 1),


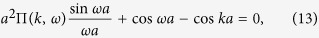


and is depicted in Fig. 2.

In Fig. 2, it shows the dispersion relation Eq. (13) from *ω* = 0 to *ω* = 5*ν*. Since Π(*K*, *ω*) (Eq. (12)) is complex, the wave number *K* satisfying the dispersion relation is also complex and will be written as *K*_*ω*_ = *k*_*ω*_ + *iκ*_*ω*_. At first, *k*_*ω*_ increases with *ω* and *κ*_*ω*_ = 0; while as *ω* is around *nν*, *k*_*ω*_ grows sharply and diminishes suddenly, and *κ*_*ω*_ grows and drops abruptly as peaks. When *K*_*ω*_ is real, it corresponds to propagating wave. While when *K*_*ω*_ is complex as *ω* ≈ *nν*, it corresponds to attenuated wave (
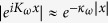
) and energy gap appears.

For monochromatic point source with frequency *ω*_*s*_ located at origin 
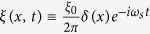
 (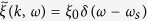), the expectation value of the photonic field at the *j*-th lattice site 〈*A(x*_*j*_, *t*)〉 is


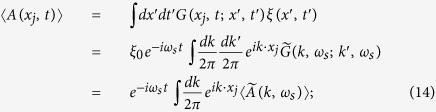


and by Eq. (29),


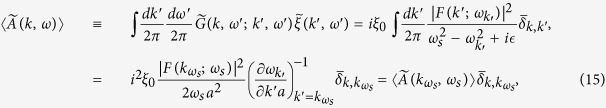


where 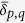 specifies crystal momentum conservation, *i*.*e*., 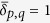, if *p* = *q* + 2*nπ*/*a*; 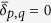, otherwise.

### DC conductivity

To discuss the transportation and conductivity of the system, it is worthwhile mentioning the following points.

**Figure 3:**
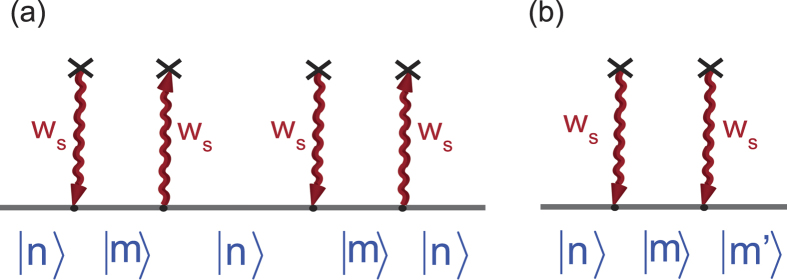
Two diagrams. In (**a**) & (**b**), the indices *n* < *N*_*F*_, and *m*′ > *m* > *N*_*F*_. (**a**) Diagram for an electron repeatedly excites above and drops below the Fermi level through continuously absorbing and emitting applied photons. (**b**) Diagram for an electron continuously absorbing more than one applied photons.

1. Our model is set at *T* = 0, and the ground state of the electrons is a fully filled Fermi sea with energy up to *ε*_*F*_.
2. We assume that the intensity of the applied EM wave is large enough, and the amplitude for spontaneous emissions of photon with frequency other than *ω*_*s*_ can be ignored.
3. By absorbing an applied photon with frequency *ω*_*s*_, an electron in the Fermi sea can be excited to a level *m* above the Fermi level *N*_*F*_ and propagate spatially; but, before reaching the end of the array, it is not allowed for an electron (with energy between *ε*_*F*_ & *ε*_*F*_ + 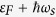) to drop to levels below *N*_*F*_ (by emitting a photon with frequency *ω*_*s*_) because they are already occupied. Thus, the diagram Fig. 3(a) is prohibited.
4. By point 2 mentioned above, electrons can stay firmly with energy below 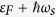; but at zero temperature, the probability to find an electron with energy higher than 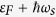 is extremely low. Thus, the situations that an electron continuously absorbing more than one applied photons and reaching levels with energy higher than 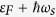 will be ignored (Fig. 3(b)) hereafter.

**Figure 4:**
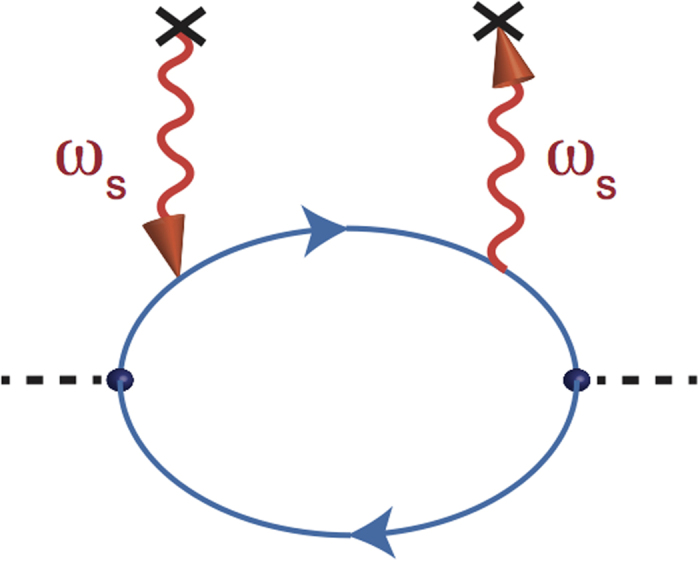
Kubo conductivity. Diagram for the calculation of the conductivity with the radiation emitted and absorbed by a source/sink (×) of photon with frequency *ω*_*s*_ to the leading order. The dotted lines each represents an insertion of *k* from the current operator.

The DC conductivity of the system can be obtained via the Kubo formula,


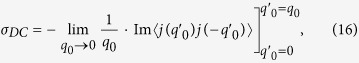


where 〈 *j(q*′_0_)*j*(−*q*′_0_)〉 is the retarded current-current correlation. Through the previous discussions of points 1–4, the leading order term (in *g*^2^|*ξ*|^2^) of the retarded current-current correlation for the arrayed SHO exposed in the EM wave at zero temperature is to calculate the diagram shown in Fig. 4,


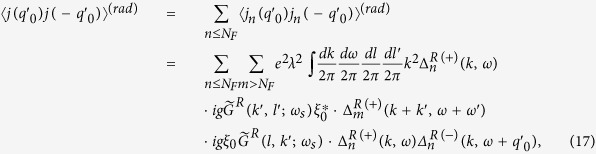


where the superscript *R* stands for the *retarded* Green functions of photon, electrons and holes.

By Eqs (16) and (17), we have the irradiated DC conductivity,


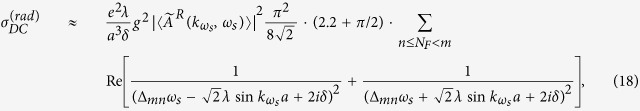


where 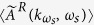 is the retarded counterpart of 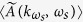 (Eq. (15)), Δ_*mn*_*ω*_*s*_ ≡ *ω*_*s*_ − (*m* − *n*)*ν*, and the above running index *m (n*) for the electron (hole) propagator should be restricted by the condition *mν* − *ε*_*F*_ < *ω*_*s*_ (*ε*_*F*_ − *nν* < *ω*_*s*_).

If there is no radiation, the retarded current-current correlation and the DC conductivity are respectively,


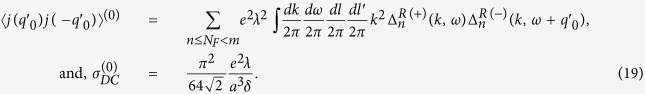


The total DC conductivity *σ*_*DC*_ is the sum of 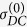 (Eq. (19)) & 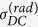 (Eq. (18)),


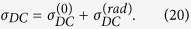


**Figure 5:**
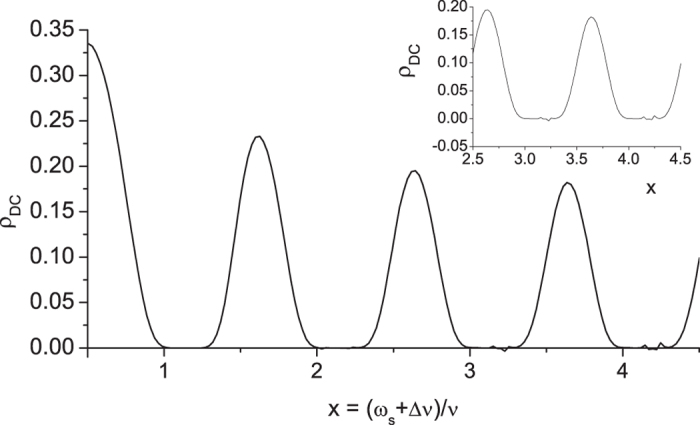
DC resistivity. DC resistivity (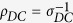) of irradiated arrayed SHO system. The vertical scale is in unit of 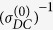. The dimensionless horizontal variable *x* is the irradiated photon energy in scale of the (renormalized) electron eigenenergy 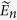. As *x* = *n* being an integer, 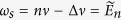, the *n*-th renormalized energy level. Here in this figure, we use the same parameters as in Fig. 2. In this case, the phase shift is around 14%. Were in two dimensions, the phase shift would then be 28%. Inset shows the detailed structure of negative *ρ*_*DC*_ as *x* is within 3 to 4.5.

The DC resistivity of our model is shown in Fig. 5 calculated from Eqs (18)–(20), 57, 60. The dimensionless horizontal variable *x* is the irradiated photon energy in scale of the (renormalized) electron eigenenergy 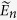. When *x* is equal to an integer *n*, the irradiated photon carries energy equal to that of the *n*-th electron eigenstate. Were in the parallel system of irradiated 2DEG in a magnetic field, that integer *n* is the quantum number of Landau level. It is demonstrated in Fig. 5 that DC resistivity has an oscillatory behavior in general. The resistivity can become zero or even negative in some regions. It also shows “phase shift” due to energy renormalization as we mentioned in the subsection of electron propagator. On the other hand, the oscillatory behavior of resistivity, zero resistance, and phase shift also appeared in many experimental works of 2d SHO systems of microwave-irradiated electron gas in a magnetic field on samples with low impurities^1^,^2^,^6^,^7^,^8^,^9^,^10^,^11^,^12^,^13^,^14^,^15^,^16^,^17^,^18^,^19^. And negative resistance appears in some experimental works on these systems^9^. Our results are in qualitative agreement with experimental works^1^,^2^. For instance, the general behavior of our DC resistivity shown in Fig. 5 is quite similar to that in Fig. 1c of ref. 1. It is worth mentioning that the horizontal variable in Fig. 1c of ref. 1, *B*^−1^/*δ* = *ω*/*ω*_*c*_, is the ratio of the incident photon energy to the cyclotron energy (the energy spacing between two adjacent Landau levels) with an integer ratio to be the quantum number of Landau level. Therefore, it is equivalent to the horizontal variable *x* defined in Fig. 5 in our work.

In addition to oscillations and zero resistance, our results also show flattened dips and phase shift. The discrepancies may come from dimensionality, impurities, and thermal phonons. This needs further investigations.

## Discussion

We studied the system of a linear array of *N* SHO interacting with a photonic field via QED interaction. Taking photon-atom interactions into account, we calculated the photonic dispersion relation. It is shown that as the energy of photon is around the spacing between SHO energy levels, the photon is absorbed and is thus in the attenuated mode. Due to many-body interactions, photons are absorbed within finite ranges of frequency. It follows that the photonic dispersion relation is modified significantly and energy gaps appear. Also the single particle wave function of photon is a Bloch wave (Eq. (25)). And these modifications are manifested in a periodic way.

The coefficient 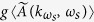 (Eqs (14) and (15)) is the amplitude of an electron absorbing a dressed photon originated from the external source and interacting with arrayed SHO through multi-scatterings. It significantly modifies the electron propagator by boosting an electron with additional momentum 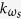 and exciting the electron to a higher excited state. Since 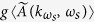 is oscillatory with respect to *ω*_*s*_, it can thus be expected that the transportation behavior of SHO array may also be oscillatory with respect to *ω*_*s*_. Therefore, the resistance of the irradiated SHO array is oscillatory (Fig. 5).

On the other hand, for the zero photo-resistance which attracts a lot of attentions, it can be seen in Fig. 2 that as *ω*_*s*_ approaches but not very close to *nν*, 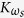 is real. For some suitable (*m*, *n*) & *λ*, it can happen that 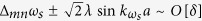; thus one of the denominators in Eq. (18) becomes very small and the irradiated conductivity is of order 
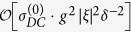
 in this case. For systems with very small *δ*, the irradiated conductivity can be very large and the corresponding resistance tends to approach zero (Fig. 5).

As to the negative resistance, it can be seen in Fig. 2, when *ω*_*s*_ is a little larger than *nν*, Re 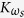 is very small and 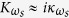. The real part of the big bracket in Eq. (18) is


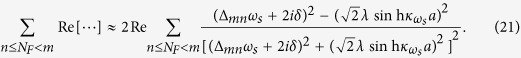


Within the range of *ω*_*s*_ mentioned above, for some suitable (*m*, *n*) & *λ*, it can happen that the RHS of the above equation is negative. Taking the parameters listed in Fig. 2, as *ω*_*s*_ = 4.027*ν*, the RHS of Eq. (21) is approximately −32*ν*^−2^, and the corresponding resistance is small but negative (*ρ*_*DC*_ = −1.746 × 10^−3^ in arbitrary unit).

In summary, we studied the QED of arrayed SHO interacting with photons. Due to multi-scatterings between photons and arrayed SHO, the photonic eigenstate is modified from a plane wave to a Bloch type wave with energy gaps in the photonic dispersion relation. The electronic states are correspondingly changed because of the close interaction between electrons and photons. Thus, the transportation behaviors of the irradiated electrons are significantly different from those with no irradiation. For instance, in addition to the oscillatory behavior, resistance can also be zero and negative. Please notice that our works investigate systems with no impurities. It can be seen that our result of photo-resistance is in qualitative agreement with experimental works on samples with relatively low impurities.

However, we believe that the mechanism in our model will be less significant as impurities in systems increase. This is because our results are closely related to the photonic dispersion relation and its behavior depends a lot on the lattice translation symmetry. As impurities increse, the lattice translation symmetry would be disturbed to some extent. On the other hand, the displacement model of the photon assisted impurity and phonon scatterings and other models with impurities involved as essential roles for electron transportation have achieved many accomplishments in systems with more impurities. We therefore tend to think that their mechanisms and ours are complimentary to each other in discussing the transportation of irradiated electronic systems with impurities. As impurities increase, their mechanisms become important; while ours gets important as impurities decrease.

In the future, we plan to apply mechanism in this work to 2DEG systems. We would also like to explore the influences of phonons and impurities to this mechanism. Moreover, the gap structure in the photonic dispersion relation we discovered will lead to significant modifications of the well-studied phenomena in different fields involving interactions between matter with lattice structure and light, such as the Dicke effect^57^,^58^ in quantum optics, the long-lived quantum memory^59^,^60^, and multiparticle quantum entanglement^61^ in quantum information processing.

## Methods

### Photonic dispersion relation *ω*_*k*_

To find the dispersion relation of the photonic field interacting with the arrayed SHO is the same as to obtain the poles of the Green’s function 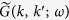, or the eigenvalues *ω*_*k*_ of the following eigenvalue equation,


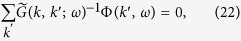


or, by Eq. (11),


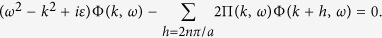


Noticing that Π(*k* + *h*, *ω*) = Π(*k*, *ω*), then we have


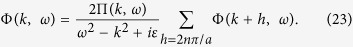


Define


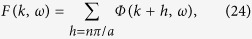


and thus *F(k* + 2*mπ*/*a*, *ω*) = *F(k*, *ω*). Please note that *F(k*, *ω*) can be chosen real and positive by multiplying a global phase to every Φ(*k* + *h*, *ω*)’s which satisfy the above equation Eq. (22).

Setting *k* as *k* + *h*′ in Eq. (23) and then summing over *h*′, we obtain,


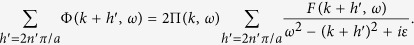


In the above equation, the LHS is exactly *F(k*, *ω*), and, on the RHS, *F(k* + *h*′,*ω*) = *F(k*, *ω*). Therefore, remembering *ε* being infinitesimal, we have,


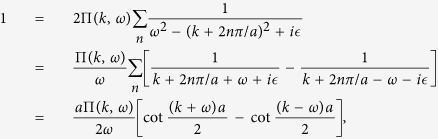


by using the identity 
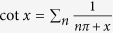
. In terms of sine and cosine functions, the above equation can be rewritten as,


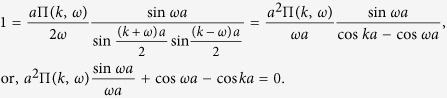


### Bloch’s wave function for photons

Due to interactions with the arrayed SHO, the eigenfunction of photon is not plane wave but the associated Bloch’s wave function. By Φ(*k*; *ω*)’s (Eq. (22)) defined previously (*k* & *ω* satisfy the on-shell relation), we can construct the spatial part of the Bloch’s wave function for photon as


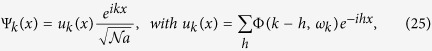


and *u*_*k*_(*x* + *a*) = *u*_*k*_(*x*). By Eq. (23) and ref. 56, the exact form of *u*_*k*_(*x*) is


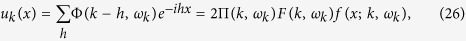


where


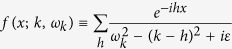



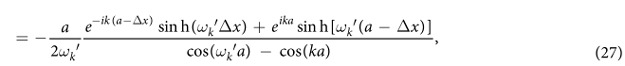


with Δ*x* = *x* − 2*π*[*x*/2*π*] ([] is the Gauss notation), and 
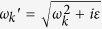
.

The normalization condition of Ψ_*k*_(*x*) requires that


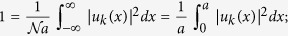


and thus it can be obtained that


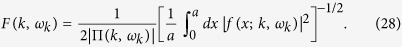


By Eq. (22), the Bloch’s states together with the electron eigenstates can form a basis in which the Hamiltonian is diagonalized. And therefore, we have


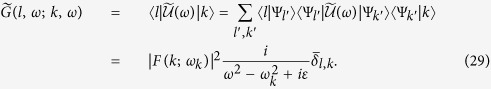


Since |Ψ_*k*′_〉 and 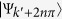 are the same, without loss of generality, we can require the Bloch state indices *k*′ and *l*′ in the above equation to be in the same Brillouin zone.

## Additional Information

**How to cite this article**: Chang, C.-C. *et al.* Dressed Photons Induced Resistance Oscillation and Zero Resistance in Arrayed Simple Harmonic Oscillators with No Impurity. *Sci. Rep.* **6**, 37763; doi: 10.1038/srep37763 (2016).

**Publisher's note:** Springer Nature remains neutral with regard to jurisdictional claims in published maps and institutional affiliations.
